# The Current and Future Role of Heart Rate Variability for Assessing and Training Compassion

**DOI:** 10.3389/fpubh.2017.00040

**Published:** 2017-03-08

**Authors:** James N. Kirby, James R. Doty, Nicola Petrocchi, Paul Gilbert

**Affiliations:** ^1^The Center for Compassion and Altruism Research and Education, Stanford University, Palo Alto, CA, USA; ^2^School of Psychology, The University of Queensland, Brisbane, QLD, Australia; ^3^John Cabot University, Rome, Italy; ^4^Centre for Compassion Research and Training, University of Derby, Derby, UK

**Keywords:** compassion, compassion interventions, heart rate variability, vagal break, evolution, compassion-focused therapy

## Abstract

The evolution of mammalian caregiving involving hormones, such as oxytocin, vasopressin, and the myelinated vagal nerve as part of the ventral parasympathetic system, enables humans to connect, co-regulate each other’s emotions and create prosociality. Compassion-based interventions draw upon a number of specific exercises and strategies to stimulate these physiological processes and create conditions of “interpersonal safeness,” thereby helping people engage with, alleviate, and prevent suffering. Hence, compassion-based approaches are connected with our evolved caring motivation and attachment and our general affiliative systems that help regulate distress. Physiologically, they are connected to activity of the vagus nerve and corresponding adaptive heart rate variability (HRV). HRV is an important physiological marker for overall health, and the body–mind connection. Therefore, there is significant value of training compassion to increase HRV and training HRV to facilitate compassion. Despite the significance of compassion in alleviating and preventing suffering, there remain difficulties in its precise assessment. HRV offers a useful form of measurement to assess and train compassion. Specific examples of what exercises can facilitate HRV and how to measure HRV will be described. This paper argues that the field of compassion science needs to move toward including HRV as a primary outcome measure in its future assessment and training, due to its connection to vagal regulatory activity, and its link to overall health and well-being.

## Introduction

Many species, such as fish, turtles, and other egg-laying reptiles produce large numbers of young ones, who need to disperse rapidly afterbirth to avoid predation, including, at times, from their own parents (1). Thus, fish and reptilian young are born to be mobile, be able to seek their own protection, and be self-sustaining. This is sometimes referred to as *r* selection. The evolution of warm-bloodedness, live birth, small numbers of young, and post birth parental/caring investment, sometimes referred to as *k* selection, required substantial changes to the physiology of threat avoidance and approach behavior, allowing for close interpersonal contact and connection. As a result, mammalian young infants did not have to be self-protective or self-sustaining, indeed there was a very different pattern of parental investment. First, parent and offspring needed to be attracted to stay close to each other rather than disperse. Second, resources and stimuli provided by the parent would have major impacts on the growth and physiological regulation of the infant (2). Third, in species where parents did provide food for offspring, in particular primates, infants are required to be quiescent for much of the time as they grow and develop (e.g., infants require lots of sleep). Fourth, because infants cannot move or provide food for themselves, the parent is responsible for providing such needs and regulating them, that is provide a *safe haven*. Fifth, as infants become more mobile, parents act as a *secure base*, facilitating gradual exploration of their environment (3).

To facilitate these interactional sequences, *k* selected regulation processors operated through a sequence of adaptations. One of these major adaptations was the evolution of part of the myelinated parasympathetic system—the dorsolateral vagal nerve that links a range of internal organs to central control systems. Indeed, the vagus nerve is connected to a range of organs including the heart and gut, and with the brain through its link to inhibitory prefrontal–subcortical circuits. One of the key functions of parental investment is sensitivity to distress and preparedness to act appropriately to relieve that distress. This is also the basic sentiment and core of compassion (4, 5), and as we will discuss, compassion utilizes the same evolved physiological pathways as basic caring behavior.

## Compassion

Compassion has been defined in various ways, with many focusing on describing certain qualities and attributes that comprise compassion (6). Other definitions stress the motivational nature of compassion, exploring its goal and focus and the various competencies necessary for that motive to operate successfully. Compassion as a motivation is central to many of the contemplative traditions (7–9). This is captured in such definitions as having *a sensitivity to suffering in self and others, with a commitment to alleviate and prevent it* (5). One way to better focus future research in compassion science is *via* evolutionary insights into its origins and functioning situated within its neurophysiological architecture (4, 5, 10, 11).

Along with compassion there are many human motives including self-protection (harm-avoidance), sexual (finding a mate and reproducing), and caring-based motives (5, 12, 13). All motives have two basic processes, which when applied to compassion include (1) having a motive-appropriate signal detection (input) to suffering (i.e., sensitivity and awareness of distress) and (2) having a behavior–output repertoire that allows appropriate responsiveness to suffering (i.e., taking action to alleviate and prevent suffering).

## Compassion and Physiology

It is now well recognized that a key process that assists with affect regulation is through caring affiliative and affectionate behaviors. Polyvagal theory, outlined by Porges (14), details how the activation of the myelinated parasympathetic nervous system helps in the regulation of the fight/flight response (autonomic sympathetic nervous system), thus enabling calmness and soothing to be achieved through having close proximity to others, giving/receiving affiliative, caring, and prosocial behavior (5, 15, 16). This is reflected in the dynamic balancing of the sympathetic and parasympathetic nervous systems that give rise to variability in heart rate [Heart Rate Variability (HRV); (14)]. In fact, the autonomic nervous system enables emotion-related action tendencies, which, in the case of compassion, are approach and caregiving. The inhibition of heart rate through the activity of the parasympathetic nervous system has shown to be linked to the orienting response and sustained outward attention, which constitute a core action tendency of compassion (17). Consistently, compassion-evoking stimuli (videos of other’s suffering) have shown to generate vagally mediated heart rate deceleration in children (18) and in adults, whose self-reports of sympathy and compassion were positively related to heart rate deceleration (19). Moreover, children with higher heart rate deceleration during evocative films showed increased subsequent compassionate behavior (20). Interestingly, children with higher baseline HRV were rated by teachers and parents as more helpful and more able to regulate their emotions than those with lower HRV (21) and showed increased self-reports of sympathy, both dispositionally and in response to distress-inducing films (22). This suggests that tonic HRV might represent the physiological signature of a trait-like compassionate responding. Increased HRV in adults has been found to be specifically connected to the emotional state of compassion and not to positive affect in general (23), supporting the correlation between vagal activity and state-like episodes of compassion. Moreover, recent findings (24) have shown that increases in vagally mediated HRV induced by Transcranial Direct Current Stimulation over the left temporal lobe, topographically close to the left insular cortex, was associated with increases in soothing positive affect: a feeling of perceived safeness and warmth which has been considered the emotional underpinning of a compassionate motivation. Feeling safe is linked to HRV, and higher HRV is linked to a greater ability to self-soothe when stressed (14), thus facilitating engagement with the suffering (in one selves and others), while inhibiting the distress-related tendencies to fight with or withdraw from suffering.

Specific strategies, such as breathing practices, friendly voice tones, and facial and body expressions, can activate the parasympathetic system, aiming to calm and soothe the individual, which improves HRV (25). Moreover, when the sympathetic nervous system is activated under threat, this decreases the ability for higher order cognitive capacities, such as mentalizing to occur (e.g., theory of mind, empathizing, perspective taking), whereas activating the parasympathetic system helps provide a feeling of safeness, which increases the ability to activate the prefrontal cortex and enable mentalization (26–28). Thus, the focus on activating affiliative processing systems (e.g., parasympathetic system) assists in the regulation of affect, and helps calm individuals when distressed. HRV, as a marker of increased emotion regulation which facilitates, and is facilitated by, an approach motivation to suffering, might be considered one of the primary measures when assessing and training compassion. Measuring HRV in compassion science is uncommon, yet there are many important nuances that need to be teased out and examined between HRV and compassion, thus more data are required to help improve understanding the interaction and precision of links between compassion and HRV.

## The Benefits of Compassion

There is now considerable evidence that being the giver and recipient of caring behaviors, particularly compassion, has a range of health benefits (29, 30) and can affect genetic expression (31, 32). Compassion training improves general well-being and social relationships (33, 34), with increasing evidence of its effectiveness as a psychotherapy (11, 35, 36). A recent review of several compassion-based interventions (e.g., compassion-focused therapy, compassion cultivation training) found moderate effect sizes for reducing symptoms of depression, anxiety, and stress, as well as increasing individuals’ levels of compassion, mindfulness, and well-being (11). Practicing compassion also has an impact on neurophysiology due to neuroplasticity (27), with a recent study showing that it has significant impacts on HRV (Matos et al., under review). What is required now is a more coordinated and integrated research program on the physiological changes that are brought about through compassion training.

## Current State of Compassion Training and Problems of Assessment

There are various approaches to compassion training, with different approaches having different definitions and consequently different forms of assessment (11). The assessment methods used in compassion training relies predominantly on self-report measures, such as the Self-Compassion Scale (37), and a recent review that critically examined the assessment measures for compassion concluded that self-report questionnaires assessing compassion have serious psychometric weaknesses (6). Thus, other assessment options need to be considered. One such approach is through physiological assessment; however, for this to be an appropriate assessment, it needs to align with the model of compassion used by the intervention. All the available compassion interventions are secular in design, however, theoretically these interventions have been typically influenced by Tibetan Buddhist traditions (11), and as such the definitions of compassion in the majority of compassion interventions do not include links to evolutionary models or physiology (11). Compassion-focused therapy is notably different to the other interventions, as the theoretical underpinning is based on an evolutionary model that links to physiology (11). Thus, assessing HRV as an assessment would be appropriate for that model, as one could interpret the assessment outcomes to its model of intervention.

## Preliminary Research Investigating HRV and Compassion

Moving beyond the use of the limited reliability of self-reports or more complex fMRI, HRV is a relatively easy measure to acquire and offers windows on a number of important physiological systems including the frontal cortex (38) and people’s relative state of psychological flexibility. The value of using HRV both as a process/state and outcome measure is linked to three major domains. First, that psychopathology (depression, anxiety, paranoia) and underlying processes, such as self-criticism, negative rumination, shame, and worry, are linked to lower levels of HRV. Second, that compassion is correlated to HRV. Third, compassion-based practices can directly increase HRV and potentially other biological, physiological, and neurophysiological measures, such as cortisol, and diminish the expression of proteins associated with inflammation. We will look at these in turn.

To date, there have been several cross-sectional studies demonstrating lower levels of HRV in conditions, such as depression (39), anxiety (40), rumination (41), and self-criticism (42). Second, there have been some correlational studies demonstrating the links between compassion and HRV. For example, trait self-compassion, measured by the Self-Compassion Scale (37) was found to be correlated to resting HRV and an increase in parasympathetic nervous system tone (43).

Third, experimental studies have also documented the impacts of compassion on physiology. For example, Rockliff and colleagues (42) examined the impact of compassionate imagery (i.e., imagine compassion coming from an external source, human or non-human) on HRV. In this study, HRV was derived by inter-beat interval time series of electrocardiography and CMetx software (44). Results were mixed with some participants having a reduction in HRV, while others showed increased HRV in response to compassion imagery. Individuals who experienced decreases in HRV in response to compassionate imagery were those that had lower levels of social safeness, and had higher scores on self-criticism, self-coldness, anxious attachment, and psychopathologies. Results indicate that compassion is linked to HRV; however, depending on an individual’s levels of self-criticalness and insecure attachment style compassionate imagery may be perceived as a threat, which decreases HRV. A second study (45) including 105 undergraduate college women (Mean age = 19.53) found that participants who had received self-compassion training (following audio guided recordings of loving-kindness and self-compassion phrases) had smaller reductions in HRV before and after exposure to a psychosocial stressor as compared to two control groups (an attention control—reading a psychology textbook or no intervention). Another study (46) found compassion meditation increased positive emotions and HRV, and that effect moderated by baseline vagal tone. Finally, a recent study by Petrocchi et al. (47) found that compassionate self-talk increased HRV and soothing positive affect, and that the effects on both physiological and self-report measures were amplified when participants were asked to repeat the phrases in front of a mirror. A time domain measure of HRV (root mean square successive differences) were obtained using HRV Analysis Software (48). These results provide further evidence for the impact of compassionate-based exercises in activating the soothing affect system connected with parasympathetic nervous system activity, and influencing HRV.

## How HRV is Used Presently in Compassion Training

There are now a number of compassion-based interventions that are aimed to improve well-being and social relationships, such as compassion cultivation training, cognitively based compassion training, mindful self-compassion, cultivating emotional balance, compassionate mind training, and others (28). Each of these approaches have been examined in randomized controlled trials, with early findings indicating compassion-based interventions are a promising intervention to help increase well-being and reduce suffering (28). Importantly, the only compassion training intervention model that has used HRV to date is compassion-focused therapy (CFT). As mentioned CFT (5), has its theoretical underpinnings based upon evolutionary psychology and attachment theory, which is linked to physiological systems, which makes assessing HRV in its model suitable (5).

To date, CFT is the only psychotherapy to actively target physiological processes in therapy; with a recent randomized controlled trial including 117 participants showed that it was able to increase HRV for participants (Matos et al., under review). Participants in the CFT intervention received online recordings covering: (a) body grounding and soothing rhythm breathing (five breaths per minute), mindfulness, compassion postures, facial expressions, and voice tones; (b) practising oneself as a compassionate person; (c) practising receiving compassion and care from another sentient caring mind; (d) working compassionately with self-criticism; and (e) bringing one’s compassionate self into everyday life and its difficulties. The intervention components described were included as they specifically focused on increasing compassion related competences and affiliative emotions which are specifically indexed by increased vagal tone and HRV [Matos et al., under review; (14)]. Compared to the control group the CFT condition experienced significant increases in: positive emotions associated with feeling relaxed and also safe and content; self-compassion, compassion for others and compassion from others. There were also significant reductions in shame, self-criticism, depression, and stress. Importantly, only the CFT condition reported significant improvement in HRV.

## The Future of Compassion Interventions and HRV

The findings from the recent RCT on CFT for HRV outcomes are encouraging (Matos et al., under review); however, experimental studies are also required to determine which specific practices and for what duration influences HRV. Exercises that can help activate HRV include focusing and practicing breathing, facial expressions, posture, and visualization strategies (4, 49). Another key practice to examine is the inner vocal tone of the client, if one’s self-talk is hostile and aggressive this can impact threat-based physiology, whereas if one’s inner self-talk is encouraging this has the potential to activate the affiliative/soothing emotional system (14). Thus, each of these specific practices require experimental testing to determine if each contributes to increasing HRV. However, insecure attachment and social safeness need to be explored as potential moderators of these effect.

If other compassion training models wish to use HRV as an assessment outcome, theoretical considerations need to be made to evolutionary models and physiological systems. For example, if other compassion interventions begin to use HRV as an outcome there will be potential difficulties in understanding and interpreting the data if it does not link to a model of compassion which links to physiological systems. Although other compassion interventions do incorporate a number of different body grounding exercises this is often with the direct aim to reduce suffering, without specific theoretical links made to physiological shifts and dynamic balancing of the sympathetic/parasympathetic systems and vagal tone (28). Thus, with the increasing use of HRV as a physiological proxy for measuring compassion, other models might begin to become more inclusive of evolutionary and physiological models to understanding compassion.

An alternative approach to cultivating compassion to increase HRV, is to target HRV directly. Recent technologies aimed at providing biofeedback in order to increase HRV are also showing promise (50), although researchers are suggesting that technologies used to increase HRV are potentially more effective when combined with mindfulness or compassion-based techniques (50). Importantly, what would be interesting in future research is to examine whether targeting increases in HRV alone can lead to increases in compassion motivation toward self and others, particularly behaviorally.

Finally, researchers examining HRV in compassion science need to consider the complexity of HRV and its dynamic relationship with vagal tone and emotion regulation. There can be a tendency as more researchers in the field of compassion science begin to assess HRV to refer to it as being a singular outcome, with higher HRV only related to positive outcomes. Indeed, there are times when high sympathetic tone is desired, for example, when helping to target states of helplessness (14). Therefore, greater precision is needed when reporting on HRV, for example if using five-minute protocols of HRV it is important to report the time of day it was assessed and for how long, which would be different when compared to reporting on 24 hour HRV protocols. Furthermore, in CFT a focus of intervention is to help improve safeness for the client, and although this is hypothesized to generally be associated with higher HRV, it may also help a client feel empowered and give them confidence to challenge themselves, which could be linked to a higher resting sympathetic tone. These nuances between HRV, vagal tone, and compassion need to reported and examined with precision to better understand the relationship.

## Conclusion

The fact that HRV is an output from the cardiovascular system is due to the evolution of the mammalian prosocial and caring behavior which has become highly tuned in humans. A range of physical and mental health conditions are linked to the degree to which people feel a sense of safeness and connected in the social environments, and HRV is increasingly considered a marker of those physical and mental states. New therapies are developing to directly influence and increase feeling of safeness, interconnection, and compassionate motivation, and HRV should be considered a primary outcome for research exploring the efficacy of those novel psychotherapy approaches.

## Author Contributions

JK, JD, NP, and PG all contributed to this topic and writing equally.

## Conflict of Interest Statement

The authors declare that the research was conducted in the absence of any commercial or financial relationships that could be construed as a potential conflict of interest.
